# Reference value of knee position sense in weight-bearing and non-weight-bearing conditions

**DOI:** 10.1186/s43019-023-00199-x

**Published:** 2023-11-27

**Authors:** Yubin Lee, Chaegil Lim

**Affiliations:** 1 Department of Health Science, Gachon University Graduate School, Incheon, 21936 South Korea; 2 Department of Physical Therapy, College of Health Science, Gachon University, 191, Hambakmo-ro, Yeonsu-gu, Incheon, 21936 South Korea

**Keywords:** Knee, Proprioception, Joint position sense, Weight-bearing, Age

## Abstract

**Background:**

Our study aimed to identify age-related changes in knee proprioception to provide reference values for weight-bearing (WB) and non-weight-bearing (NWB) conditions and to identify factors (age, WB condition, dominance, and sex) that can affect knee proprioception.

**Methods:**

A total of 84 healthy adult men and women were recruited. Active knee joint position sense (JPS) was measured using a digital inclinometer for knee proprioception. The participants performed the required movements actively, with verbal feedback from the examiner, slowly moving to the target angles (30° and 50°) and maintaining them for 5 s before returning to the starting position. Afterward, without assistance from the examiner, the participants actively moved back to the same angle, and the examiner confirmed the angles. This procedure was repeated twice for each target angle, and the average values were used as the data. The participants were barefoot, wearing shorts, and closed their eyes while the measurements were obtained. The measurements were first obtained on the dominant side under the NWB conditions. When a change in posture was needed during the measurement, the participants sat in a resting position for 2 min.

**Results:**

Except for age, all other factors (WB condition, dominance, sex) were not statistically significant. Age showed a significant difference in knee JPS, except for the non-dominant side at 30° and the dominant side at 50° in the NWB condition.

**Conclusion:**

This study indicates that the WB condition, dominant side, and sex need not be considered when measuring and assessing knee JPS. Age shows a negative correlation with knee joint position sense, and the reference values presented in this study can be used as objective target values during the rehabilitation process.

## Introduction

The knee joint is crucial in supporting substantial body weight [1, 2]. Various bones (femur, patella, tibia, and fibula), joints (medial, lateral, and proximal tibiofemoral joints), muscles, tendons, and ligaments responsible for complex functions related to stability and mobility connect to the knee joint [3, 4]. Proprioception involves nerve input to the central nervous system through different mechanoreceptors [3–6]; it plays a vital role in the somatosensory system [1, 7]. Knee proprioception is essential for everyday activities such as walking and sports, enabling us to perceive and control body movement, position, overall coordination, and joint ability [8]. Impaired proprioception results in increased body sway, reduced balance, and a higher risk of falling, potentially leading to further injuries [9, 10].

Knee proprioception is currently evaluated by various methods such as the weight load [weight-bearing (WB), partial WB, or non-WB (NWB)] [6, 7], measuring equipment (goniometer, inclinometer, Biodex, or Cybex) [6, 10–12], the method of joint reposition (supine, prone, sitting, or one leg squat) [10, 13, 14], and the target angle types [1, 14, 15].

These various methods have led to a lack of standardized criteria, and functional improvement has been primarily assessed through before and after intervention comparisons. Regarding the weight load factor, most studies on proprioception have emphasized WB as a more accurate measurement method. However, situations where full knee extension is not possible due to anterior cruciate ligament reconstruction [11], other balance issues [13, 16, 17] and other conditions necessitate measurements in the NWB condition.

The primary objective of this study is to establish reference values for knee proprioception in the WB and NWB conditions, providing target benchmarks for rehabilitation. Additionally, we hypothesized that other factors (age, WB condition, dominance, and sex) influence proprioceptive sensitivity, warranting the need for reference values that consider these factors.

## Methods

### Participants

A total of 84 participants were recruited on the basis of an effect size of 0.55, alpha of 0.05, and beta of 0.80. This study was approved by the Gachon University Institutional Review Board (approval number 1044396-202303-HR-037-01) and the Clinical Research Information Service (no. KCT0008518). Informed consent was obtained from all participants before the start of the study.

Because osteoarthritis, which can affect the knee proprioception, is found in 30–50% of adults > 65 years old and at least one joint in >80% of older people, individuals aged between 20 and 50 years were recruited [18].

The exclusion criteria were as follows: (1) history of surgery on the lower extremities, (2) neurological or musculoskeletal impairment, (3) limitation of active and passive knee joint motion, and (4) discomfort or pain while performing the required measurements.

### Knee proprioception measurement

The measurement of knee proprioception has been substituted with the assessment of joint position sense (JPS). The active knee joint repositioning test was performed under WB and NWB conditions. The measurements were initially taken in the NWB position, followed by 2 min in a seated position before subsequent measurements were obtained in the WB position. The participants were barefoot, wore shorts, and closed their eyes to avoid visual cues while the measurements were obtained.

All participants rested in a chair with a backrest for 2 min before measurement. To determine the dominant leg at rest, the examiner asked each participant which foot was used to kick a ball when it rolled toward them. The participants actively and slowly moved to the target angle from the starting position until the examiner said “stop.”

The target angle was maintained at this position for 5 s. The participants actively returned to the starting position following the instructions of the examiner. After, the examiner instructed the participant to move again as much as they just moved. The participants actively repositioned their knee and maintained this position for 3 s. At this point, the examiner confirmed the angle. The target angles were randomly set to two different values (30°and 50°), and each angle was measured twice. A resting period of 3 s was taken in the initial position for each trial. The average value of two trials was used for the analysis.

Following this procedure, the dominant side (DS) was first measured, and after a 2 min rest in the sitting position, the opposite leg was measured. The difference between the measured and target angles was used as the absolute angular error (AAE) value without directional bias. A digital inclinometer (AOSYCO, Miami, FL, USA) was used to verify the angles. The same examiner obtained all measurements without assistance (e.g., touch feedback from the examiner, auditory feedback) or encouragement.

#### Weight-bearing condition

**Fig. 1:**
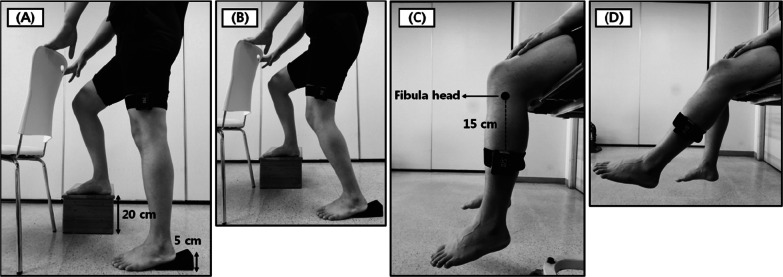
Initial and measurement positions for knee joint position sense. **A** Start position in weight-bearing condition; **B** measurement posture in weight-bearing condition; **C** start position in non-weight-bearing condition; and **D** measurement posture in non-weight-bearing condition

The weight-bearing measurement was conducted by referring to previous studies on posture [19, 20]. The starting position was a one-leg standing position with the measurement leg supporting the body. A 5-cm high footboard was used on the heel of the measurement leg to reduce the passive tension on the calf muscle (Fig. 1A). A 20-cm high step box was used to slightly flex knee and hip joints and to relax the opposite leg (Fig. 1A). A chair in front of the participant was used to maintain minimum balance. The digital inclinometer was placed on the lateral side at the distal one-third of the length from the anterior superior iliac spine to the patella of the measurement leg (Fig. 1A). Based on previous studies, a pilot experiment was performed by placing a digital goniometer on the anterior thigh. However, this placement method resulted in instability and inaccurate measurements. For improved accuracy, the digital inclinometer was configured in the following manner: target angles of 30° and 50° were selected, considering findings from prior research [12, 19]. The angle of 30°, within the range of 20–40°, is highly correlated with proprioception feedback during regular walking, making it more precise for functional measurements. The 50° angle represents the midpoint of knee flexion, where adjustments by the muscle sensor play a dominant role in detecting knee joint position. The participants performed eight trials {both legs [DS, non-DS (NDS)] × 2 target angles [30°, 50°] × 2 trials}. In addition, during the test, the participants were supervised at a close distance to prevent falls.

#### Non-weight-bearing condition

**Fig. 2:**
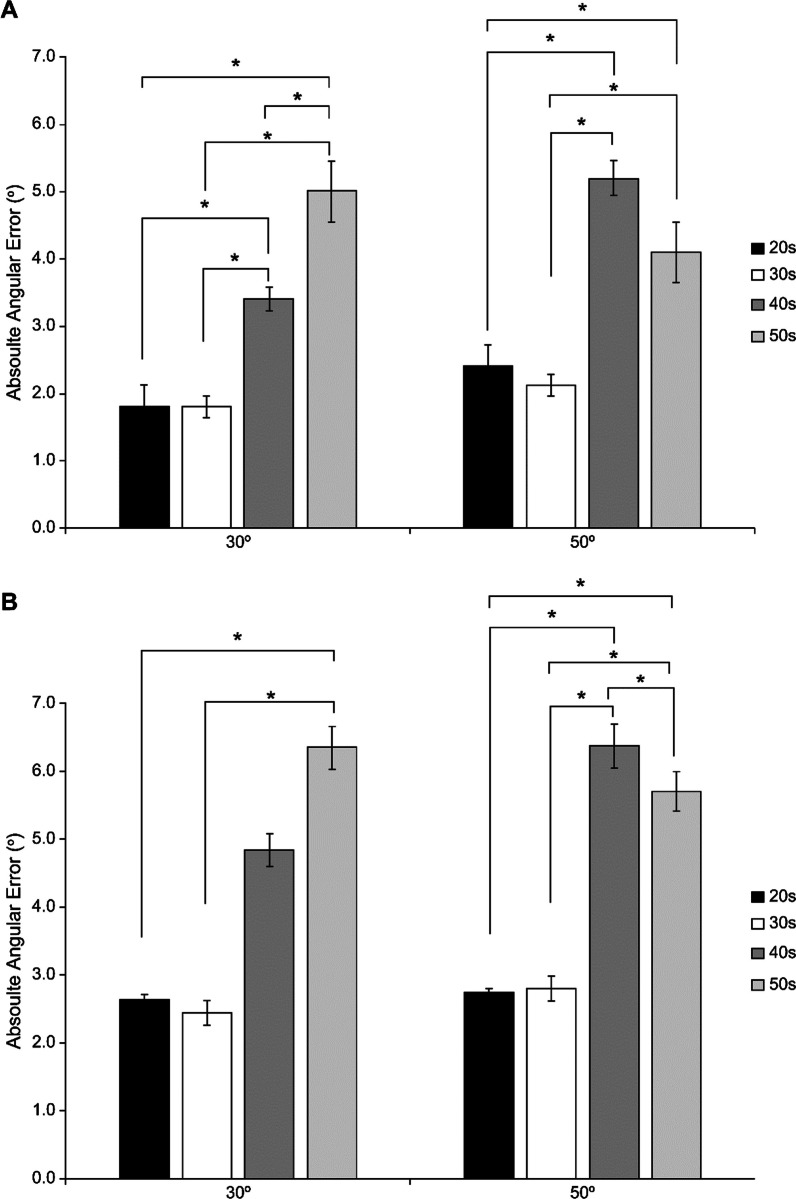
Comparison of knee joint position sense between groups (20 s, 30 s, 40 s, and 50 s). **A** Weight-bearing condition; **B** non-weight-bearing condition

The non-weight-bearing measurement was conducted with reference to previous studies on posture [20, 21]. The starting position was a neutral sitting position with both hands placed on the knee (Fig. 1C). The digital inclinometer was placed on the lateral side, 15 cm below the fibula head, using a black strap with a width of 1.8 cm (Fig. 1C). Measurements were conducted from knee flexion to knee extension. The knee extension position should be measured as the starting position in the NWB condition to ensure consistent measurements with the WB condition. However, because of the quadriceps muscle activation involvement, which prevents complete NWB, we performed the measurements in the opposite direction. The target angles of 30° and 50° were chosen for consistency with the WB condition. Each participant completed eight trials, encompassing both legs (DS, NDS) and target angles (30° and 50°), with two trials conducted for each combination (Fig. 2).

### Statistical analysis

Statistical analyses were performed using SPSS ver. 25.0 (SPSS Corp., Armonk, NY, USA). The averages and standard deviations were calculated for all variables. Normal distribution for all variables was confirmed using the Shapiro–Wilk test. A simple regression analysis evaluated the relationship between AAE and age. An independent *t*-test was used to confirm the differences between AAE and identify the age, WB condition, dominance, and sex factors. A *P* value of < 0.05 indicated statistical significance.

## Results

All participants had a dominant right leg. The average knee flexion angle in the NWB condition was 93.2° ± 5.66°. The characteristics of each age are presented in Table 1. Except for age, all other factors (WB condition, dominance, sex) were found to be statistically insignificant in all variables, indicating that these factors did not have a significant impact on knee proprioception (Table 2). The only factor that impacted on knee proprioception was age factor, which positively correlated with AAE in all variables except for the NDS 30° and DS 50° in the NWB condition (Table 3).

**Table 1:** The characteristics of the participants

Variable	Age (years)	Height (cm)	Weight (kg)	BMI (kg/m^2^)	Knee flexion angle (°)
20 s (*n* = 21)	25.3 ± 1.70	169.9 ± 7.99	64.0 ± 10.84	22.0 ± 2.07	94.8 ± 8.00
30 s (*n* = 21)	33.1 ± 2.69	169.5 ± 9.31	69.3 ± 13.62	23.9 ± 2.90	92.2 ± 6.15
40 s (*n* = 21)	45.1 ± 2.91	169.1 ± 8.69	67.7 ± 14.74	23.2 ± 4.02	92.8 ± 4.00
50 s (*n* = 21)	54.8 ± 2.81	167.4 ± 7.16	68.5 ± 9.62	24.4 ± 2.19	92.9 ± 4.89
Total (*n* = 84)	39.5 ± 11.60	169.2 ± 8.09	67.7 ± 12.10	23.4 ± 3.05	93.2 ± 5.66

Values are presented as mean ± standard deviation

*BMI* body mass index

**Table 2:** Relationship between the knee joint position sense and identify factors (weight bearing conditions, dominance differences, and sex differences)

Variable			Target angle (°)	AAE (mean ± SD)	*P*^1^	*P*^2^
WB	Sex	Male	30	3.32 ± 3.63	0.104	0.082
			50	3.41 ± 3.20		
		Female	30	4.15 ± 4.25		
			50	4.99 ± 4.47		
	Dominance	DS	30	3.75 ± 4.11	0.456	
			50	4.55 ± 4.23		
		NDS	30	3.66 ± 3.80		
			50	3.88 ± 3.41		
NWB	Sex	Male	30	3.39 ± 3.13	0.878	
			50	3.74 ± 3.41		
		Female	30	3.43 ± 3.04		
			50	3.63 ± 2.75		
	Dominance	DS	30	3.62 ± 3.23	0.170	
			50	3.96 ± 3.39		
		NDS	30	3.19 ± 2.93		
			50	3.47 ± 2.72		

*AAE* absolute angular error, *WB* weight-bearing, *NWB* non-weight-bearing, *DS* dominant side, *NDS* non-dominant side

*P*^1^ < 0.05, compared with the parameters of different sex and different dominance

*P*^2^ < 0.05, compared with the parameters of different weight-bearing conditions

**Table 3:** Relationship between the age and the knee joint position sense

Variable	*R*^2^	Constant	*β*	Standard error	*P﻿*
WD 30	0.47	−2.775	0.166	0.036	0.000*
WND 30	0.42	−1.767	0.137	0.034	0.000*
WD 50	0.39	−1.070	0.141	0.039	0.000*
WND 50	0.42	−1.219	0.126	0.032	0.000*
NWD 30	0.37	−0.419	0.102	0.030	0.001*
NWND 30	0.18	1.412	0.045	0.029	0.122
NWD 50	0.15	2.207	0.044	0.033	0.188
NWND 50	0.33	0.447	0.076	0.026	0.004*

*WD 30* weight-bearing + dominant side + 30°, *WND 30* weight-bearing + non-dominant side + 30°, *WD 50* weight-bearing + dominant side + 50°, *WND 50* weight-bearing + non-dominant side + 50°, *NWD 30* non-weight-bearing + dominant side + 30°, *NWND 30* non-weight-bearing + non-dominant side + 30°, *NWD 50* non-weight-bearing + dominant side + 50°, *NWND 50* non-weight-bearing + non-dominant side + 50°

*Significant difference (*P* < 0.05)

For each age group, we provide the reference values for knee proprioception under both WB and NWB conditions in Table 4.

**Table 4:** Reference value of knee joint position sense on age

Age	Variable	AAE (mean ± SD)	
		30°	50°
20–29 (*n* = 21)	WD	1.53 ± 1.67	2.55 ± 2.50
	WND	2.13 ± 1.69	2.29 ± 1.98
	NWD	2.03 ± 2.26	3.05 ± 2.78
	NWND	3.29 ± 3.03	2.45 ± 1.96
30–39 (*n* = 21)	WD	1.76 ± 1.40	2.50 ± 1.29
	WND	1.84 ± 1.78	1.74 ± 1.88
	NWD	2.79 ± 2.82	3.05 ± 2.69
	NWND	2.11 ± 2.09	2.55 ± 2.31
40–49 (*n* = 21)	WD	4.18 ± 2.59	5.92 ± 3.85
	WND	2.61 ± 2.45	4.42 ± 2.95
	NWD	5.90 ± 4.37	7.10 ± 4.64
	NWND	3.80 ± 2.95	5.63 ± 3.22
50–59 (*n* = 21)	WD	5.38 ± 4.02	3.83 ± 3.51
	WND	4.68 ± 3.45	4.40 ± 3.01
	NWD	5.95 ± 5.07	5.95 ± 5.17
	NWND	6.73 ± 5.30	5.45 ± 4.40

*AAE* absolute angular error, *WD* weight-bearing + dominant side, *WND* weight-bearing + non-dominant side, *NWD* non-weight-bearing + dominant side, *NWND* non-weight-bearing + non-dominant side

## Discussion

This study aimed to investigate the reference values of knee proprioception based on active knee JPS in WB and NWB conditions and to identify the factors that may influence JPS. The results contradicted the hypothesis of all identified factors significantly impacting JPS and requiring a reference value accordingly. The JPS was only found to be associated with age. Regarding the relationship between age and JPS, AAE increased with age under the WB conditions, indicating a decline in JPS ability. However, in the NWB condition, the measured angle only affected the JPS on the DS for the smaller angle (30°), whereas for the larger angle (50°), it only influenced the JPS on the NDS. In other words, in the NWB condition, age was affected by the angle and dominance difference.

Existing research has attributed the age-related decline in proprioception to alterations in the muscle spindle functionality [22, 23]. Although the number of motor units decreases in older individuals, compensatory hypertrophy and slowed reorganization can affect the proprioception of integrated sensory and motor information [24, 25]. Aging causes various functional changes, including motor, sensory, and cognitive changes and changes in fat and body mass index [8, 24, 26]. Ultimately, the overall functional decline associated with aging can have a detrimental effect on JPS. This is supported by the results of this study, showing that the AAE was significantly smaller in those in their 20s than in those in their 40s and 50s. Therefore, we suggest that the reference values provided in this study can be used clinically as target values for each age group.

This study examined the relationship between the WB condition and JPS and found that the WB condition showed lower AAE values, suggesting greater accuracy, but there was no significant difference. Refshauge and Fitzpatrick [27] reported that a neutral sitting position with knee flexion and slight ankle plantarflexion in the NWB position had a perception threshold for a movement twice as high as that in the standing position in the WB position. This suggests that calf muscle stretching on the basis of foot and knee postures plays an important role. In addition, Wise et al. [28] reported that the contraction of the surrounding muscles influenced the threshold for position detection. Herrington [7], Magalhães et al. [19], Stillman and McMeeken [29], and Vallbo [30] stated that under closed kinetic chain (CKC) conditions, sensory feedback from the surrounding joints (ankle and hip) is involved, and the eccentric contraction of knee extensor muscles is more demanding than that in open kinetc chain (OKC), resulting in the recruitment of more motor units and greater activation of muscle spindles, ultimately exerting a positive influence on knee JPS. In practical situations, it is likely impossible for patients to perform and obtain accurate values in the WB condition during the early stages of rehabilitation. Therefore, while it is possible to get accurate values in the WB condition, considering efficiency and validity, conducting assessments in the NWB condition during the initial phases of rehabilitation is recommended. As functional improvement progresses, a gradual transition to the WB condition for evaluation is suggested. However, when assessing in NWB condition, if the DS is affected, it should be evaluated at smaller angles. In contrast, when the NDS is affected, assessment at larger angles is advisable.

We hypothesized that the DS, more frequently used in daily activities, would exhibit stronger connections between motor and sensory functions in a higher overall interaction among brain regions and smaller AAE values. However, interestingly, the NDS showed smaller AAE values than the DS, and this difference was not statistically significant. Hoshiyama and Kakigi [31] observed that only the contralateral hemisphere responded when the dominant hand was used, whereas both hemispheres responded when the non-dominant hand was used. They suggested that the interaction in the somatosensory cortex increases during unskilled movements with the NDS, leading to a higher somatosensory perception. Han et al. [32] examined the dominance difference in proprioception for the ankle, knee, shoulder, and fingers and consistently reported that the non-preferred side showed significantly better results across all four joints. In our study, the NDS had smaller AAE values than the DS of approximately 0.49°, indicating compensatory activation of the brain cortex in the NDS. Previous studies on dominance differences have reported consistent findings. Macedo and Magee [33] found no clinical range of motion differences between the DS and NDS groups in the upper and lower extremities. Schorderet et al. [34] reported that dominance difference did not affect balance performance. These findings, including those of our study, provide further evidence that dominance differences do not clinically affect overall body movement. In addition, based on the results of this study, it is implied that it is acceptable to perform the assessment using the same method without considering the dominant leg of the patient. When considering only the dominance factor, using the measurement values of the opposite leg as the reference point is permissible. However, it is important to note that in this case, although to a minor extent, the non-dominant leg tends to have more accurate values.

Bulut and Pehlivan [35] reported that physical activity level is related to body awareness, with men showing a higher level of physical activity than women. Edwards [36] conducted a sensory interaction and balance clinical test using the Biodex system in healthy adults in their 20s and found that men exhibited approximately 23% smaller body sway than women. In contrast, Puszczalowska-Lizis et al. [37] conducted a stabilographic test on adults aged 60–90 years and found that men had lower postural stability in the mediolateral and anteroposterior directions than women. Based on these studies, we assumed that there would be sex differences. However, our study indicates that sex differences did not impact the JPS. Bryant et al. [38] reported no significant differences in static balance between men and women among healthy adults aged 50–60 years. Harrington [7] and Ghiasi and Akbari [6] found differences in knee proprioception between men and women under OKC and CKC conditions, but these differences did not significantly impact them. Similar to previous studies, this study does not explain the causes of sex differences. However, it suggests no sex differences in the JPS, indicating the absence of sex differences in posture perception and balance ability. Instead, individual performance should be considered.

This study has several limitations. First, the participants performed eight repetitions per leg during measurements between sessions, making it impossible to ignore the carryover effect. Second, the positions of the pelvis and trunk of the participants were not standardized during the measurements. Third, owing to the age limit of 50 years set for the participants, we could not establish reference values for knee proprioception for individuals aged ≥ 60 years. In future research, reference values on knee proprioception for individuals aged ≥ 60 years should be obtained. Moreover, conducting the study in a manner that considers the positioning of the trunk and pelvis while also implementing methods to minimize carryover effects is essential.

## Conclusions

This study investigated the reference values for knee JPS according to age. Additionally, we examined whether age, WB condition, dominance, and sex affected knee JPS. This study suggests that other variables, excluding age, need not be considered when measuring and evaluating knee JPS. The age factor exhibits a negative correlation with knee JPS, and the reference values presented in this study can be utilized as objective target values during rehabilitation. Under the NWB condition, it is necessary to set the DS to a larger angle and the NDS to a smaller angle for implementation.

## Data Availability

The datasets generated and/or analyzed during the current study are not publicly available due to the potential risk of individual privacy exposure, but are available from the corresponding author upon reasonable request.

## References

[1] Bennell KL, Hinman RS, Metcalf BR, Crossley KM, Buchbinder R, Smith M, McColl G (2003). Relationship of knee joint proprioception to pain and disability in individuals with knee osteoarthritis. J Orthop Res.

[2] Relph N (2015). The measurement of knee joint position sense.

[3] Carpenter JE, Blasier RB, Pellizzon GG (1998). The effects of muscle fatigue on shoulder joint position sense. Am J Sports Med.

[4] Voight ML, Hardin JA, Blackburn TA, Tippett S, Canner GC (1996). The effects of muscle fatigue on and the relationship of arm dominance to shoulder proprioception. J Orthop Sports Phys Ther.

[5] Riemann BL, Lephart SM (2002). The sensorimotor system, part I: the physiologic basis of functional joint stability. J Athl Train.

[6] Ghiasi F, Akbari A (2007). Comparison of the effects of open and closed kinematic chain and different target position on the knee joint position sense. J Med Sci.

[7] Herrington L (2005). Knee-joint position sense: the relationship between open and closed kinetic chain tests. J Sport rehabil.

[8] Ferlinc A, Fabiani E, Velnar T, Gradisnik L (2019). The importance and role of proprioception in the elderly: a short review. Mater Sociomed.

[9] Blasier RB, Carpenter JE, Huston LJ (1994). Shoulder proprioception: effect of joint laxity, joint position, and direction of motion. Orthop Rev.

[10] Bullock-Saxton JE, Wong WJ, Hogan N (2001). The influence of age on weight-bearing joint reposition sense of the knee. Exp Brain Res.

[11] Mesfar W, Shirazi-Adl A (2008). Computational biomechanics of knee joint in open kinetic chain extension exercises. Comput Methods Biomech Biomed Engin.

[12] Suner-Keklik S, Cobanoglu-Seven G, Kafa N, Ugurlu M, Guzel NA (2017). The validity and reliability of knee proprioception measurement performed with inclinometer in different positions. J Sport Rehabil.

[13] Bang DH, Shin WS, Choi SJ, Choi HS (2015). Comparison of the effect of weight-bearing and non-weight-bearing positions on knee position sense in patients with chronic stroke. J Phys Ther Sci.

[14] Drouin JM, Houglum PA, Perrin DH, Gansneder BM (2003). Weight-bearing and non-weight-bearing knee-joint reposition sense and functional performance. J Sport Rehabil.

[15] Kaynak H, Altun M, Tok S (2020). Effect of force sense to active joint position sense and relationships between active joint position sense, force sense, jumping and muscle strength. J Mot Behav.

[16] Barcellona MG, Morrissey MC (2016). The effect of open kinetic chain knee extensor resistance training at different training loads on anterior knee laxity in the uninjured. Man Ther.

[17] Piriyaprasarth P, Morris ME, Delany C, Winter A, Finch S (2009). Trials needed to assess knee proprioception following stroke. Physiothera Res Int.

[18] Loeser RF (2010). Age-related changes in the musculoskeletal system and the development of osteoarthritis. Clin Geriatr Med.

[19] Magalhães T, Ribeiro F, Pinheiro A, Oliveira J (2010). Warming-up before sporting activity improves knee position sense. Phys Ther Sport.

[20] Romero-Franco N, Montaño-Munuera JA, Jiménez-Reyes P (2017). Validity and reliability of a digital inclinometer to assess knee joint-position sense in a closed kinetic chain. J Sport Rehabil.

[21] Saeed Alshahrani M, Reddy RS, Asiri F, Tedla JS, Alshahrani A, Kandakurti PK, Kakaraparthi VN (2022). Correlation and comparison of quadriceps endurance and knee joint position sense in individuals with and without unilateral knee osteoarthritis. BMC Musculoskelet Disord.

[22] Hashizume K, Kanda K (1995). Differential effects of aging on motoneurons and peripheral nerves innervating the hindlimb and forelimb muscles of rats. Neurosci Res.

[23] Verdú E, Ceballos D, Vilches JJ, Navarro X (2000). Influence of aging on peripheral nerve function and regeneration. J Peripher Nerv Syst.

[24] Ribeiro F, Oliveira J (2007). Aging effects on joint proprioception: the role of physical activity in proprioception preservation. Eur Rev Aging Phys Act.

[25] Brown WF (1972). A method for estimating the number of motor units in thenar muscles and the changes in motor unit count with ageing. J Neurol Neurosurg Psychiatry.

[26] Lee YS, Nichols JF, Domingo A, Kim Y, Park SM, Han G, Seo H, Hovell M (2021). Balance performance and related soft tissue components across three age groups. Health Care Women Int.

[27] Refshauge KM, Fitzpatrick RC (1995). Perception of movement at the human ankle: effects of leg position. J Physiol.

[28] Wise AK, Gregory JE, Proske U (1999). The responses of muscle spindles to small, slow movements in passive muscle and during fusimotor activity. Brain Res.

[29] Stillman BC, McMeeken JM (2001). The role of weightbearing in the clinical assessment of knee joint position sense. Aust J Physiother.

[30] Vallbo AB (1974). Human muscle spindle discharge during isometric voluntary contractions: amplitude relations between spindle frequency and torque. Acta Physiol Scand.

[31] Hoshiyama M, Kakigi R (1999). Changes of somatosensory evoked potentials during writing with the dominant and non-dominant hands. Brain Res.

[32] Han J, Anson J, Waddington G, Adams R (2013). Proprioceptive performance of bilateral upper and lower limb joints: side-general and site-specific effects. Exp Brain Res.

[33] Macedo LG, Magee DJ (2008). Differences in range of motion between dominant and nondominant sides of upper and lower extremities. J Manip Physiol Ther.

[34] Schorderet C, Hilfiker R, Allet L (2021). The role of the dominant leg while assessing balance performance: a systematic review and meta-analysis. Gait Posture.

[35] Bulut N, Pehlivan E (2023). Does physical activity level depend on exercise perception and body awarness?. Türk Fizyoterapi ve Rehabilitasyon Dergisi.

[36] Edwards HM (2011). Gender differences in balance of college-aged students.

[37] Puszczalowska-Lizis E, Bujas P, Jandzis S, Omorczyk J, Zak M (2018). Inter-gender differences of balance indicators in persons 60–90 years of age. Clin Interv Aging.

[38] Bryant EC, Trew ME, Bruce AM, Kuisma RM, Smith AW (2005). Gender differences in balance performance at the time of retirement. Clin Biomech.

